# Supplementary material for: A set of multi-entry identification keys to African frugivorous flies (Diptera, Tephritidae)
Source: Zookeys. 2014 Jul 24;(428):97–108. doi: 10.3897/zookeys.428.7366 (PMC4143993; doi:10.3897/zookeys.428.7366)
Supplement: Supplementary material 6 — Key to Ceratitis [file zookeys-428-097-s006.zip › SF6_ZooKeys_key to Ceratitis/key/SF6_ZooKeys_key to Ceratitis/Media/Html/Ceratitis pedestris .htm]

Ceratitis (Pterandrus) pedestris (Bezzi)


***Ceratitis*** **(*Pterandrus*) *pedestris*
(Bezzi)**

*Pardalaspis pedestris* Bezzi, 1924a: 480.

Body
length: 4.55 (4.20-5.05) mm; wing length: 4.73 (4.30-5.30) mm.

Male

Head: Antenna yellowish orange. First flagellomere twice as
long as pedicel. Arista with short rays; basoventral rays shorter and sparser
than basodorsal rays. Frons convex or flat, yellow (sometimes more palish);
with short scattered setulae distinctly darker than frons. Frontal setae
slightly underdeveloped. Face yellowish white. Genal seta and setulae dark,
well developed. Occiput on dorsal part yellowish brown to brown colored except
along dorsal margin; not always distinct.

Thorax:
Postpronotal lobe white, with black spot (sometimes more brownish). Scutal
pattern: ground color brownish black, sometimes orange-tinged (especially in
West African specimens), microtrichose areas silvery with ashgray shine, spots
brownish black or black, rarely spots reduced and orange ground color
extensive; prescutellar yellow-white markings merged or separate. Scapular setae
dark. Scutellum yellow, basally with two separate dark spots, apically with
three separate dark spots, extending to basal 0.25. Anepisternum completely
pale, at most ventral margin very narrowly yellow; with pale setulae except few
dark setulae along dorsal and ventral margins.

Legs:
Yellow except where otherwise noted; setation typical for subgenus, mainly
pale. Foreleg: coxa anteriorly shiny black with silvery spot; femur with
contrasting black/white pattern, white band turns silvery when viewed from
certain angle, ventrobasally with bush of short dark setulae, continued
posteriorly as longer setulae; posteroventrally dark on basal 0.66, with bush
of white setulae in dark patch, ventral setae dark, forming small tuft
distally. Midleg: without feathering, femur with brown basal spot and few
isolated long setulae ventrally in basal part; tibia yellow with black apical
spot anterodorsally. Hindleg: femur at apical 0.25 with longer setulae dorsally
and ventrally.

Wing:
bands yellow to yellowish brown. Marginal band forming continuous band
with anterior part of discal band; cubital band free; medial band absent;
crossvein R-M opposite middle of discal cell. Apex of vein R_1_ distal
to level of crossvein R-M. Crossvein DM-Cu oblique posterobasally.

Abdomen:
Mostly dark yellow; tergite 1 with brown patches posteriorly; tergites 2 and 4
with weak silvery transverse band along� posterior half; tergite 3 with two
dark brown spots on posterior half, sometimes more extensively brown;
tergite 5 with posterior margin brown. Male epandrium in lateral view with
lateral surstylus long and slender; posterior lobe short and straight.

Female

As
male except for the following characters: Frontofacial angle sometimes with
darker patches. Frontal setae well developed. Postpronotal lobe usually with a
larger and more conspicuous spot. Anepisternum brownish on ventral 0.33. Apical
spots on scutellum touching basal spots or almost so, sometimes partly merged.
Wing bands somewhat darker than in male. Legs without feathering; yellow,
femora darkened; setation mixed pale and dark; forefemur posteroventrally with
pale pilosity. Oviscape shorter than preabdomen. Aculeus about 10 times longer
than wide; tip very slender, pointed and lateral margin distinctly concave.

(Description
after De Meyer & Freidberg, 2006)
